# A Case Report of Spontaneous Pneumomediastinum (Hamman's Syndrome) During Labor

**DOI:** 10.7759/cureus.75944

**Published:** 2024-12-18

**Authors:** Christopher Barrera-Hoffmann, Yadira Mariaca-Ortíz, Mauro Alejandro Rodríguez-Flota, Paul Alejandro Cerda-García, Enrique Monares-Zepeda

**Affiliations:** 1 Critical Care Medicine, Instituto Mexicano del Seguro Social, Juarez, MEX; 2 Nephrology, Instituto Nacional de Cardiologia Ignacio Chávez, Mexico City, MEX; 3 Emergency, Hospital General Regional 1, Mérida, MEX; 4 Critical Care, Hospital General Regional 1, Mérida, MEX; 5 Critical Care Medicine, Coordinación de Terapia Intensiva y Hemodinamia de Hospitales de 2º Nivel Instituto Mexicano del Seguro Social Bienestar, Mexico City, MEX

**Keywords:** hamman's syndrome, labor, obstetrics, pneumomediastinum, valsalva maneuver

## Abstract

Hamman's syndrome is characterized by spontaneous pneumomediastinum triggered by Valsalva maneuvers and is an uncommon complication during labor and the postpartum period. It is typically benign and managed conservatively with oxygen therapy and analgesia. We present the clinical case of a 21-year-old primigravida who developed spontaneous pneumomediastinum during labor, manifesting with subcutaneous emphysema and dyspnea. It was documented by a chest X-ray and CT scan, and improved with conservative treatment. The patient was discharged without complications or recurrence. Hamman's syndrome is a rare condition, so recognizing it accurately is fundamental. This allows for timely treatment initiation, detection of complications, and exclusion of other pathologies, all of which affect patient prognosis.

## Introduction

Hamman's syndrome is a condition characterized by the occurrence of spontaneous pneumomediastinum, defined as the presence of air in the mediastinum without an apparent precipitating cause. It is a rare and typically benign condition that responds to conservative management and was first described in the literature by Louis Hamman in 1939 [1-3].

Its pathophysiological explanation results from alveolar rupture, secondary to the pressure gradient between the pulmonary interstitium and the alveoli, usually due to the Valsalva maneuver. The escaping air then flows through the pressure gradient toward the hilum and subsequently into the mediastinum [4,5].

It can present as primary spontaneous pneumomediastinum but has also been associated with certain pulmonary conditions, such as asthma, COPD, interstitial lung diseases, and bronchiectasis [6]. This can be due to precipitating events that include coughing, sneezing, vomiting, defecation, and labor [1,7].

The incidence during pregnancy is rare, occurring in approximately one out of every 100,000 vaginal deliveries, primarily in young primiparas at term [8]. Up to 55% of the reported cases occur during the prolonged second stage of labor, and it also appears in the immediate postpartum period in 16.5% of cases [2,9].

We present the clinical case of a 21-year-old primigravida who experienced spontaneous pneumomediastinum due to the Valsalva maneuver during labor. We have preserved the anonymity of the patient who provided informed consent.

## Case presentation

We present the case of a 21-year-old female primigravida patient who was 36.3 weeks pregnant based on the calculations from the first-trimester ultrasound. Her personal pathological history included no chronic illnesses, allergies, transfusions, traumatic events, or previous hospitalizations. The pregnancy was a planned one and the patient had been receiving prenatal care since the first trimester, including six consultations. The pregnancy had progressed normally, with regular intake of folic acid and iron supplements.

She presented to the obstetric emergency department of General Regional Hospital due to regular pelvic contractions and obstetric pain lasting 12 hours. She denied any vaginal bleeding or warning symptoms. Upon assessment at the first contact, vital signs were within normal ranges along with an adequate fetal heart rate. An obstetric ultrasound evaluation revealed that the amniotic fluid and placenta were adequate. During the gynecological examination, the pelvis and conditions favorable for vaginal delivery were assessed. The patient was in spontaneous labor at the initial evaluation and was admitted at 14:45 h for active labor. She delivered a male infant vaginally at 17:00 h, weighing 2970 grams, measuring 50 cm, Capurro 40 weeks of gestation, and an APGAR score of 8/9, with a blood loss of 150 ml. The cord and placenta were normal. Immediate skin-to-skin contact and breastfeeding were initiated.

During the third phase of labor, the patient presented with discomfort and subcutaneous emphysema in the cervical region extending to the mandibular region, along with dyspnea that improved with the administration of conventional supplemental oxygen. Owing to the suspicion of a critical illness, the patient was evaluated by the intensive care team, who reported subcutaneous emphysema on the right hemiface, anterior neck, and thorax, along with a slight increase in respiratory effort and a decrease in oxygen saturation at 90%, measured by pulse oximetry. This improved with supplemental oxygen support to 98%, with a respiratory rate of 24 breaths per minute, a heart rate of 94 beats per minute, and a blood pressure of 110/60 mmHg. Upon physical examination, the lung fields revealed adequate entry and exit in both hemithoraces, left-sided tympanic percussion, increased heart sounds with good intensity, a globular abdomen with an involuted uterus up to the umbilical scar, and extremities without clinical edema and with immediate capillary refill.

**Figure 1:**
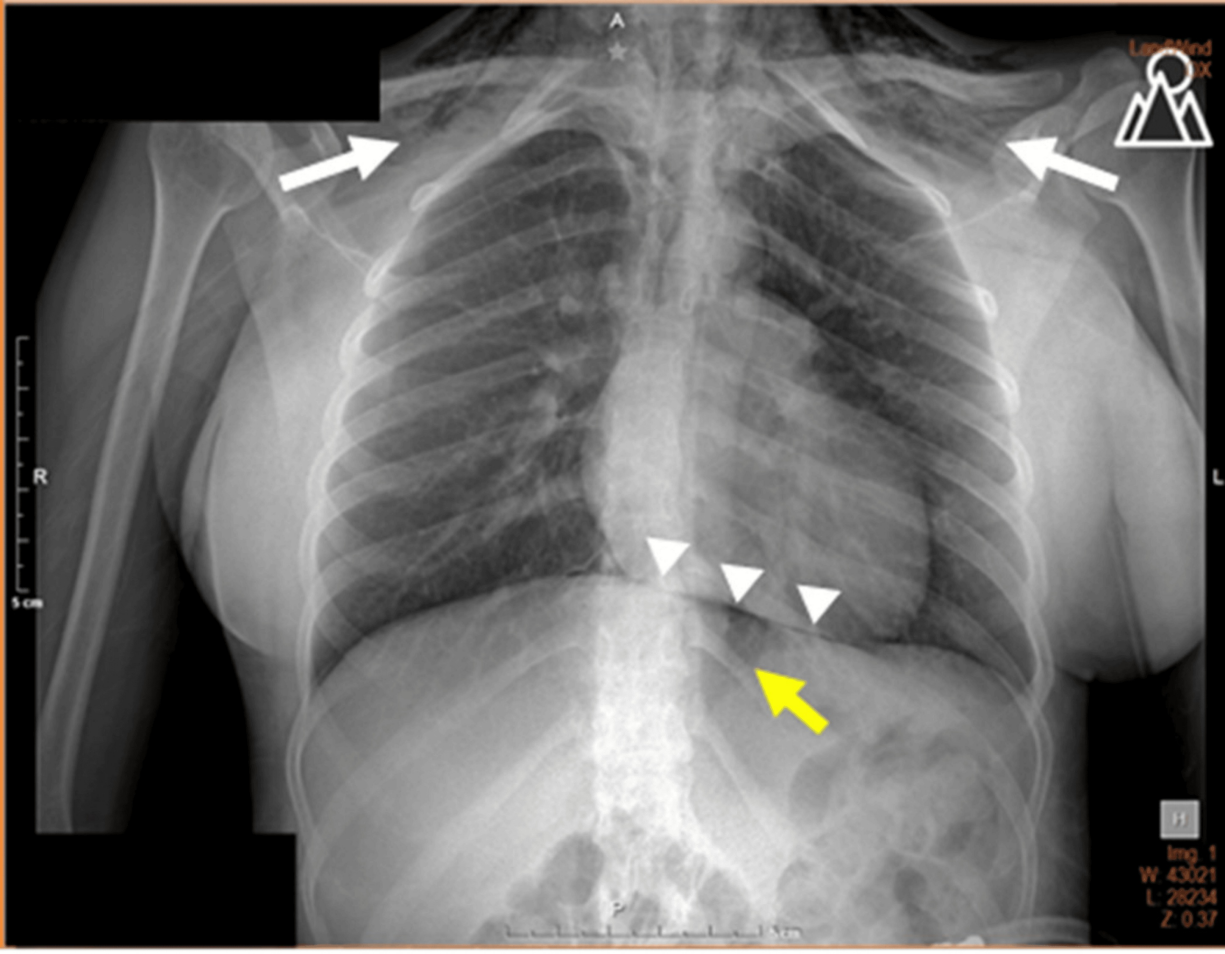
Anteroposterior chest X-ray on day zero The image shows subcutaneous emphysema in the clavicular regions extending to the neck (white arrows), with radiographic signs of pneumomediastinum, including Naclerio’s V sign (yellow arrow) and the continuous diaphragm sign (white arrowheads).

The chest X-ray revealed subcutaneous emphysema in the soft tissues of the neck, a central air column, preserved costodiaphragmatic recesses, no separation of the parenchyma from the thoracic cage, and signs of pneumomediastinum (Figure 1).

Signs of pneumothorax were ruled out. However, on the basis of the described data, admission to the intensive care unit was decided upon, where analgesia and oxygen therapy was continued. The respiratory and hemodynamic status was monitored and a plain chest CT scan was requested to assess the extent of pneumomediastinum.

**Figure 2:**
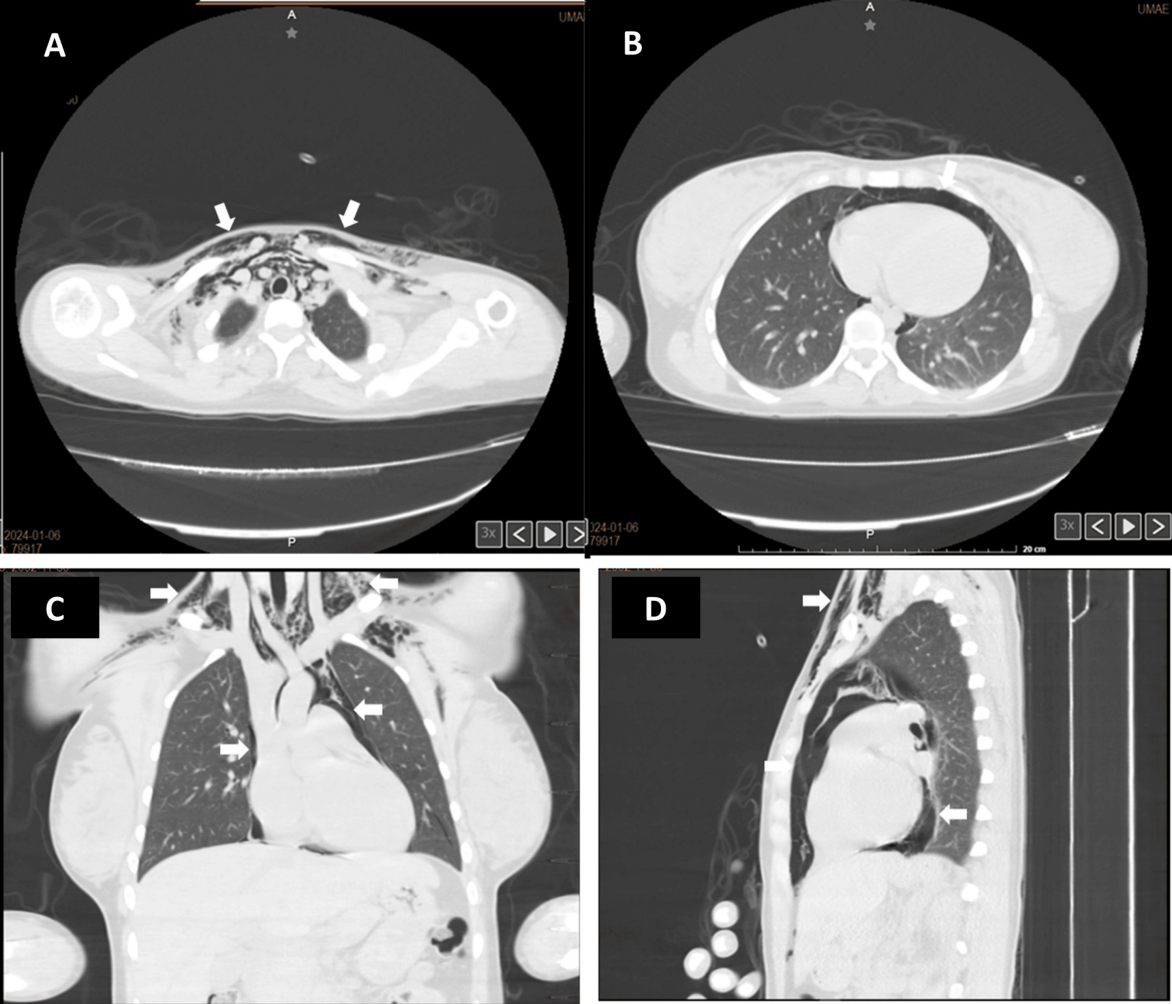
Non-contrast chest CT (lung window) on day one Different planes, i.e., axial (A and B), coronal (C), and sagittal (D) showing subcutaneous emphysema extending to the soft tissue of the supraclavicular region and neck (A and C) and in the anterior sternal region (D) along with evidence of pneumomediastinum (B, C and D). CT: computed tomography

The plain chest CT scan (Figure 2) revealed soft tissues with the presence of subcutaneous emphysema dissecting the soft tissues of the neck, vascular structures, and subcutaneous cellular tissue in the supraclavicular region, originating from the mediastinum, with a separation of 14 mm from the anterior chest wall to the heart. No signs of pneumothorax or pleural dissection were identified, and no apparent lesions of the upper gastrointestinal tract were observed through this imaging method.

**Figure 3:**
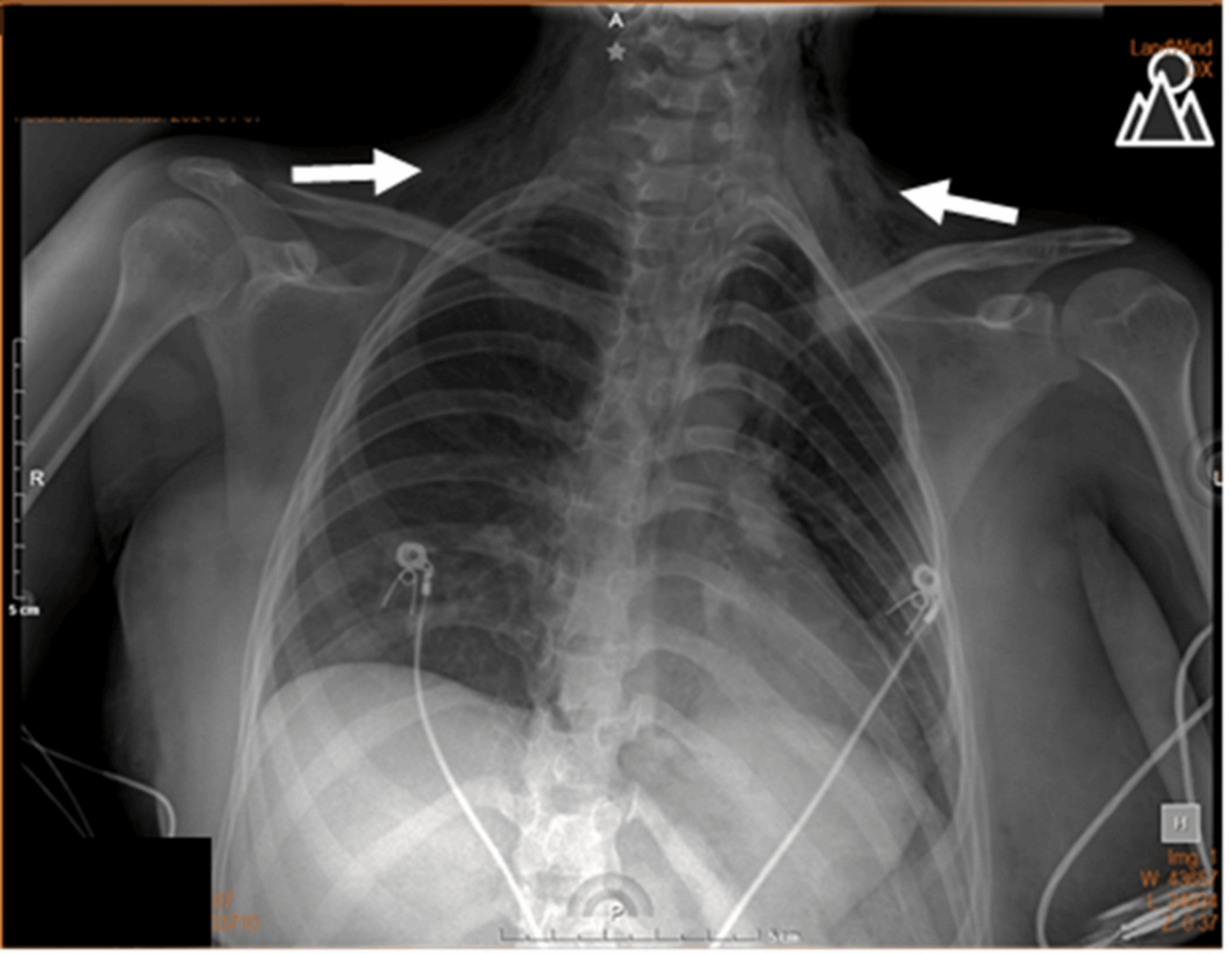
Anteroposterior chest X-ray on day two The image reveals a reduction in the subcutaneous emphysema in the clavicular and cervical regions (white arrows).

During her stay in the intensive care unit, the patient continued with conventional oxygen therapy via a low-flow nasal cannula and analgesics. A follow-up radiographic study was performed on the second day, which revealed a decrease in subcutaneous emphysema, with no other radiographic findings of pneumomediastinum (Figure 3).

Supplemental oxygen was continued with a gradual withdrawal on day three. There was a reduction in subcutaneous emphysema in the neck and thorax along with an improvement in the initial symptoms. Throughout her stay, the patient did not present any alarming signs or hemodynamic instability. So, on the third day, she was discharged from the ICU and was monitored in the obstetric ward during the postpartum period. She shared a room with her newborn for breastfeeding and maternal bonding. There was no recurrence of symptoms or subcutaneous emphysema, and after two days in the ward, she was discharged from the hospital.

## Discussion

In spontaneous pneumomediastinum, the clinical presentation is characterized by a sudden onset of symptoms, the most common being chest pain, dyspnea, dysphonia, odynophagia, and dysphagia, in addition to signs such as edema and subcutaneous emphysema. Furthermore, a small percentage of patients may exhibit Hamman's sign (mediastinal crunch that is heard with each heartbeat) [5,7,10]. Typically, the described symptoms resolve on their own without additional problems and rarely recur, even in subsequent pregnancies [11,12].

Usually, a chest X-ray, which has a sensitivity of 91-95%, is used and can help detect up to 70% of cases. However, in cases where the pneumomediastinum is too small to be seen on the X-ray, a chest CT scan may be useful to rule out any underlying causes [1,11]. The diagnosis is confirmed by clinical findings, physical signs, and radiological evidence of the presence of air in the mediastinum, as well as the absence of other specific pathological causes present in secondary pneumomediastinum [1].

The occurrence of associated complications is not common; however, an infrequent complication is tension pneumomediastinum (also described as malignant pneumomediastinum), which leads to high mediastinal pressures that can cause obstructive shock, requiring urgent management and surgical intervention [8,9].

The differential diagnosis aims to identify pathologies that present with pneumomediastinum and mimic some of the symptoms, some of which may pose greater severity and risk for the patient. Therefore, spontaneous esophageal perforation (Boerhaave syndrome) must be ruled out, as it is precipitated by the same factors and presents with pneumomediastinum, accompanied by Mackler's triad: vomiting, chest pain, and subcutaneous emphysema [4]. Other differential diagnoses to consider, based on the clinical context, include acute coronary syndrome, pericarditis, aortic dissection, pulmonary embolism, amniotic fluid embolism, pneumothorax, and rupture of the tracheobronchial tree [2,13].

Once the diagnosis is confirmed, treatment can be initiated. However, there is no consensus on the management of this condition, so the treatment approach varies. It can involve oxygen therapy, analgesia, and bed rest, as well as emotional support to reassure the patient, including the need for anxiolysis [8,9,13]. There is no indication of dietary restriction, and early feeding can be initiated, preventing nutritional deficits that could result in a reduced ability to breastfeed and affect the early maternal-infant bonding [1,14].

The relief of symptoms and the radiographic resolution of pneumomediastinum are determining factors for assessing the patient's discharge, with follow-up not being necessary in the vast majority of cases [1].

## Conclusions

Hamman’s syndrome is a rare condition that can occur during labor and in the postpartum period, typically presenting as a benign and self-limiting condition with a low incidence of complications. Its management is usually conservative and expectant. However, due to its potential association with increased maternal morbidity and mortality, particularly in young postpartum patients, it is critical to remain vigilant and consider differential diagnoses that may worsen the clinical outcome. Preventive measures are essential for patients with known risk factors, such as excessive vomiting or asthma. These include avoiding prolonged or intense Valsalva maneuvers during delivery, considering epidural analgesia to minimize the risk of active pushing, utilizing assisted delivery with forceps or vacuum, or opting for a cesarean section in cases of difficult or prolonged labor. Early recognition, appropriate management, and careful consideration of risk factors are essential to ensure optimal outcomes for both the mother and the newborn.
